# Sustainable Operation and Maintenance Modeling and Application of Building Infrastructures Combined with Digital Twin Framework

**DOI:** 10.3390/s23094182

**Published:** 2023-04-22

**Authors:** Zedong Jiao, Xiuli Du, Zhansheng Liu, Liang Liu, Zhe Sun, Guoliang Shi

**Affiliations:** 1 Faculty of Architecture, Civil and Transportation Engineering, Beijing University of Technology, Beijing 100124, China; jiaozedong01@163.com (Z.J.); duxiuli@bjut.edu.cn (X.D.); liulang@bjut.edu.cn (L.L.); zhesun@bjut.edu.cn (Z.S.); guoliangshi@emails.bjut.edu.cn (G.S.); 2 The Key Laboratory of Urban Security and Disaster Engineering of the Ministry of Education, Beijing University of Technology, Beijing 100124, China

**Keywords:** digital twin, building infrastructures, sustainable operation and maintenance, energy consumption prediction, event backtracking

## Abstract

Sustainable management is a challenging task for large building infrastructures due to the uncertainties associated with daily events as well as the vast yet isolated functionalities. To improve the situation, a sustainable digital twin (DT) model of operation and maintenance for building infrastructures, termed SDTOM-BI, is proposed in this paper. The proposed approach is able to identify critical factors during the in-service phase and achieve sustainable operation and maintenance for building infrastructures: (1) by expanding the traditional ‘factor-energy consumption’ to three parts of ‘factor-event-energy consumption’, which enables the model to backtrack the energy consumption-related factors based on the relevance of the impact of random events; (2) by combining with the Bayesian network (BN) and random forest (RF) in order to make the correlation between factors and results more clear and forecasts more accurate. Finally, the application is illustrated and verified by the application in a real-world gymnasium.

## 1. Introduction

The operation and maintenance of in-service buildings has a history of more than 40 years. The notions of ‘operation’ and ‘maintenance’ first appeared in 1976 [1], addressing the issues of large apartment operations brought on by urbanization. With growing populations and continuous economic improvement, the form and functionality of large-scale buildings have become more diverse and complex, accompanied by people’s definitions of high-quality lifestyles constantly evolving and the promotion of more convenient and effective means of operation and maintenance. In the early days, only manual management and service were provided. The data of each aspect of the building can now though be acquired via sensors with the development of technology. The collected data can be used to assist analysis and decision-making, leading to more convenient and intelligent operation and maintenance for both users and managers. The advances in integrating big data technology with the operation and maintenance of buildings are enabled by the following aspects: (a) the identification of the key influencing factors of the target building using the coupling relationship model of big data technology to rationally use energy [2]; (b) the prediction and detection of changes in the external environment, timely determination of energy utilization [3], reduction in unnecessary equipment energy consumption, and improvement of staff efficiency [4]; (c) the intelligent monitoring of equipment abnormalities [5] and extension of building service life [6] through intelligent effective operation and maintenance; (d) the intelligent advocation and deduction of events affecting operation and maintenance, as well as an increase in profitability for commercial operation venues such as stadiums.

Moreover, people have become increasingly concerned with environmental issues. Buildings, as a high energy-consuming body, have become the focus of people’s attention. The activity and people flow in buildings are uneven. Intelligent operation and maintenance can play a significant role in ensuring the reasonable consumption of water, electricity, lighting, and air conditioning in buildings. On the basis of the original operation and maintenance, building sustainable operation and maintenance has become a new direction, from human to social needs and even social development requirements [7]. It can minimize impact on the environment while maintaining personnel comfort and building functionality. The operation and maintenance management of buildings has also been upgraded to meet the demands of intelligent operation and maintenance and includes the functions of data monitoring, data analysis, data visualization, and auxiliary decision-making. Combined with digital technology, digital operation and maintenance systems are the overall trend of the development of various industries [8]. In all kinds of buildings, large public buildings have the characteristics of a larger volume, many personnel, functional areas, high energy consumption, large amounts of equipment, easily produced operation dead angles, and difficult operation and maintenance supervision [9].

Therefore, this study focuses on large-scale public buildings. With the purpose of achieving efficient sustainable operation and maintenance, the analysis of building data is the foundation for intellectualization. Thus, comprehensive information needs to be recorded, such as history, operational records, facility performance, and exact location. Various technologies and devices (such as sensors and cameras) are adopted to collect these data [6,10]. Another complex problem is the prediction of trends [2]. Obtaining real-time data enables dynamic presentation. With the purpose of achieving prediction, it is necessary to comprehensively analyze multiple data, discover coupling relationships, and consider changing factors so as to continuously improve the accuracy of prediction [11,12]. In addition to the data that can be monitored, event factors in some daily activities can also induce significant changes in operational data that cannot be monitored by the sensor. Therefore, it is also urgent to combine the impact of events and integrate the event content into the intelligent algorithm to make a supplementary contribution to the auxiliary building detection and target result prediction, apart from developing more accurate intelligent sensing equipment to obtain data to assist the operation and maintenance management system to display dynamic information [4,13].

In 2013, Motawa, I. et al. [14] began to study building maintenance systems based on BIM, which is a combination of early and digital building operation. In 2015, Nakama, Y. et al. [15] collected and managed building information data based on Internet of Things technology. In 2017, Davtalab, O. [16] studied facility management (FM) in the life cycle of buildings. In recent years, with the comprehensive combination of building operation and maintenance and digitization, digital twin (DT) technology provides a more comprehensive solution for the intelligent operation and maintenance of buildings. The term “Digital Twin” was first coined by the American space agency (NASA) in 2010 [17]; it can copy buildings in physical space into a twin in digital space through the data of the sensor, so as to perform a visual display, that is, a data preview. According to the characteristics of large public buildings and DT technology architecture, the types of large buildings in the application process are further subdivided in this paper, and experiments are conducted with large stadiums as an example. Compared with other large buildings, the indoor air velocity, equilibrium temperature, and instantaneous flow of people also require the operation and maintenance model to have expanding ability and play a more targeted role in different buildings.

The innovation of this study is to establish a sustainable operation and maintenance DT model for large buildings based on the framework of DT, standardize the monitoring data format, improve the accuracy of building energy consumption analysis, and enhance the ability of the model to mine the influencing factors of building operation and maintenance. The Bayesian network model is introduced into the model to predict events. Then, it is combined with the random forest algorithm to predict the energy consumption results. This scheme addresses the black box (unknown) state of the process event correlation factors while relying solely on data and machine learning algorithms for prediction. In this way, the model can self-check and supplement the influencing factors and form an operation and maintenance model that can grow iteratively. The model test provides a practical reference for the application of the DT model in building intelligent operation and maintenance.

## 2. Literature Review

### 2.1. Operation Management of Building Infrastructures

Compared to the design and construction phase, operation and maintenance have a longer time span and involve many contents and complex personnel. Especially for building infrastructures, the integration of multi-functionality and the diversity of personnel significantly increase. Advanced operation and maintenance management can improve not only the overall safety of buildings but also personnel efficiency. Early building operation and maintenance primarily focused on structural safety and cost efficiency. For instance, structural performance metrics were utilized to ensure safe operation and functionality under normal conditions [18] and extreme events. Beyond the security perspective, decision-making associated with operation and maintenance stemmed from the optimization of life-cycle costs [19,20]. However, the operation and maintenance of previous studies were mainly derived from a stationary assumption, failing to capture the challenges due to the incurred information explosion and the full-space monitoring of large-scale buildings. With the recent development of the Internet of Things (IoT) and the building information model (BIM), the operation and maintenance of large-scale building gears towards a more intelligent and interactive manner [21]. Motawa et al. [14] developed a BIM-based management approach to determine information acquisition and exchange efficiency based on the collected data (such as maintenance records, work orders, and warning failures). In 2015, Nakama et al. [15] integrated building operation and maintenance with IoT to develop a more efficient way to amass building information. This method was further utilized to save energy, extend the life of buildings, improve user satisfaction, and reduce operating costs. In 2016, Che-Ghani et al. [22] studied the factors that can influence and minimize operating and maintenance costs.

In recent years, the rapid development of intelligent operation and maintenance management has benefited from the display of its effects and the emergence of various new technologies [17]. It mainly involves more comprehensive data collection and visualization technology innovation [23], as well as more abundant hardware support such as image recognition technology [24], three-dimensional point cloud modeling [25], and unmanned aerial vehicle (UAV) tilt photography [26]. The building information model combined with IoT technology facilitates the health monitoring of large building structures, realizes the regional monitoring data information corresponding to spatial visual monitoring, and quickly locates dangerous components [27]. However, BIM can only realize the dynamic visualization of models and information sharing. Hence, intelligent algorithms should be combined to achieve effective planning and lower the life-cycle cost of construction projects, resulting in the development needs of intelligent operation and maintenance [28,29].

The main experimental target of this paper is a large gymnasium. In addition to the huge volume of the gymnasium, the environmental requirements of the indoor venue are specific, and the instantaneous flow of people is large. Personnel safety and fire safety during the evacuation process need special attention [30]. The site requires several pieces of high-power equipment to support daily operation, and the large-span steel structure requires a more comprehensive building health detection system [31,32]. Meanwhile, the gymnasium is a typical large-span building, with a large roof area and abundant solar energy resources [33]. This is relatively conducive to the green management of buildings and energy conservation and carbon reduction. Stadiums are also commercial venues, and different events affect the profitability of the stadium, making them more operational than other types [34]. Despite considerable efforts to achieve smart operations, the lack of a well-organized framework/system to connect the effective management of all assets and the ability to manage the required information is one of the crucial issues in operations management. The digital twin is an intelligent digital system making full use of models and data while integrating artificial intelligence, machine learning, and data analysis in its creation. Hence, it can learn and update simulation models from multiple sources and represent and predict the current and future status of physical counterparts. The simulation model is oriented to the product life-cycle process, plays a role as a bridge and link between the physical world and the information world, and provides more real-time, efficient, and intelligent services [35].

### 2.2. Intelligent Operation and Maintenance and DT

As more and more data are collected, computer algorithms are used more frequently, generating new possibilities for operation and maintenance. Internet of Things technology, coupled with finite element software, enables the static and dynamic analysis of buildings [36,37]. Wang et al. [13] used random forest (RF) to predict hourly building energy consumption and analyze the importance of variables to determine the most influential features over different periods. Smarra et al. [38] introduced a predictive control method based on historical building data to overcome the difficulties (cost, time, and effort) related to the key factors of model predictive control (MPC) widely used in complex systems such as buildings and the identification of building prediction models. The method using machine learning algorithms such as regression trees and random forests is called data-driven model predictive control (DPC), which aims to improve robustness to uncertainties in real data collection and weather forecasting. Rivas et al. [39] adopted a Bayesian neural network (BNN) to predict remaining useful life (RUL) and its uncertainty for the effective predictive maintenance of equipment health, contributing to minimizing the cost and number of unplanned maintenance operations. Siryani et al. [40] employed a Bayesian belief network to enhance the cost efficiency of complex systems of public utility network operation and maintenance life cycle. The support technology is based on probability and data mining technology. It has a pattern detection function and can extract fault precursors by events. This predictive solution will proactively reduce maintenance costs and improve overall system management and operational efficiency, performance, reliability, and customer satisfaction.

With the explosive development of intelligent algorithms, researchers are also trying to integrate multiple data and multiple algorithms. Digital twin technology provides the possibility to achieve this goal. The digital twin is a prediction model, proposed by Tuegel et al. [41] in 2011. In the same year, the structural behavior of aircraft was determined by analyzing and simulating the behavior of aircraft from their digital model. One year later, the National Aeronautics and Space Administration (NASA) defined DT as “an integrated multi-physics, multi-scale, probabilistic simulation of a vehicle or system that uses the best physical models available, sensor updates, fleet history, and so forth, to mirror the life of its flying twin” [42,43].

From this definition, the purpose of DT can be expanded to add or extend new capabilities to physical entities by means of virtual–physical interactive feedback, data fusion analysis, and decision iterative optimization. In the field of architecture, DT technology was first used in complex product assembly workshops for real-time data acquisition, organization, and management [44]. This technology was gradually extended to all kinds of buildings, even the whole life cycle, and the popularity of keywords in the field of architecture research has changed from expert systems to BIM and DT [45].

The building information model is not always sufficient in life-cycle asset management, especially in the operational phase. To this end, a framework for the future development of intelligent asset management was proposed, integrating the concept of DT [46]. The concept of DT evolves into a comprehensive solution to manage, plan, predict, and present digital models of building/infrastructure or urban assets, which are dynamic representations of data and mimic their real behavior. Yu et al. [47] defined an extended organization method based on the COBie standard for tunnel twin data and integrated data, objects, and knowledge levels using semantic web technology. Additionally, the way assets are planned, delivered, operated, maintained, and managed has been reinvented thanks to DT’s data analysis and decision-making capabilities, providing better service [46]. The new DT-based anomaly detection process of Lu et al. [48] enabled the continuous anomaly detection of pumps. They employed a Bayesian change point detection method that processed feature data to identify and filter contextual anomalies through cross-referencing with external operational information. Wang et al. [49] proposed a sustainable building maintenance system framework GBMS (DT-GBMS) based on DT, assisting the operation team in solving the issues of the insufficient information and automation management of sustainable building maintenance.

Lu et al. [46] established a DT system architecture specifically designed for building and city levels. Following this architecture, a DT Demonstrator for the West Cambridge site of the University of Cambridge, UK, was developed. The demonstrator integrates heterogeneous data sources, supports effective data query and analysis as well as decision-making processes in operational management, and further bridges the gap between people and buildings/cities.

However, the machine learning algorithm has a black box problem. The association process between metadata and results is unknown, and the algorithm itself has been relatively fixed without significant improvement. It is difficult to break through the fixed range by relying solely on data and algorithms to predict the results. Concurrently, more and more related technologies have led to the disunity of data in the quantity and format of each operation and maintenance side, making it not conducive to the iterative upgrading of products in the industry. If there is a standard or unified framework, intelligent operation and maintenance will have a faster development speed in the research and market application fields. Hence, this study aims to further explore the relationship between factors and prediction results and enable the model to self-regulate the associated factors according to different scenarios.

### 2.3. Sustainable Operations

With the support of multi-functional sensors and intelligent algorithms, researchers have gradually expanded the goal of intelligent operation and maintenance. In recent years, in addition to the building itself and its users, the relationship between the building and the external environment, which is also related to the economic and sustainable operation and maintenance of the building, has attracted increasing attention.

**Figure 1:**
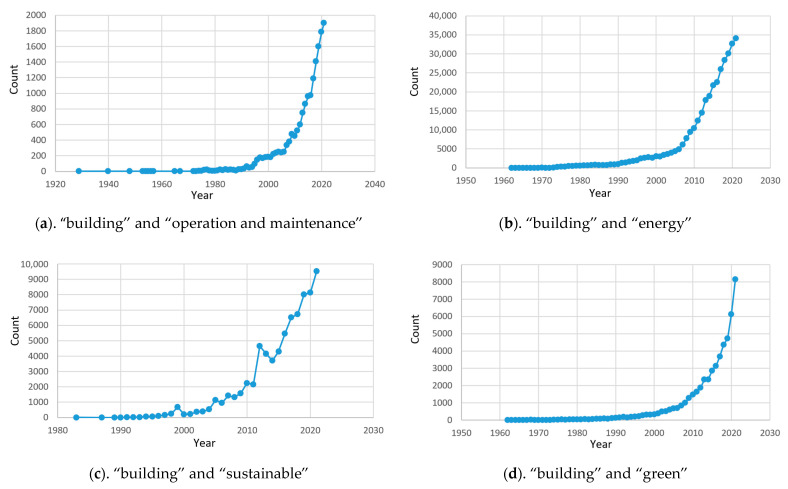
Change curve of the number of documents published by different keywords on Web of Science.

After years of hard negotiations, the ‘Paris Agreement’ to wrestle with climate change was finally reached in 2015. Countries are cooperating to limit the global temperature rise to 2 °C compared to before the industrial revolution by the end of this century and striving not to exceed 1.5 °C. However, the scenario analysis of global greenhouse gas emissions in the IPCC’s 2021 report demonstrates that even in the best-case scenario, global warming is expected to peak at 1.6 °C by mid-century when the world reaches net zero emissions by 2050. Global warming over 1.5 °C will have catastrophic effects on human society. This is a major challenge. In terms of policy, many countries have also successively set carbon neutrality targets and formulated relevant regulations. Concerning energy consumption, the whole process of building accounts for 50.6% of national carbon emissions and 46.5% of national energy consumption, according to the statistics of the China Building Energy Conservation Association. Building, as a high energy consumption body, is deeply concerning for society. Increasingly, researchers have begun to conduct relevant research on the green and sustainable operation and maintenance of large public buildings. From one perspective, more practical structures and materials will be adopted to improve the reliability of buildings and reduce maintenance costs. From another perspective, energy conservation and emission reduction will be achieved by using green materials and formulating energy conservation strategies. Research on the sustainability and intelligent operation and maintenance of buildings is increasing year by year, as shown in Figure 1.

To achieve the coordinated and sustainable development of architecture and the environment, Jensen et al. [50] maximized the reliability and availability of bridges and minimized costs, environmental impacts, and the potential for hazards through bridge operations from the planning and design stages. Kim et al. [51] maximized the expected service life of the building and minimized the expected life-cycle cost, including inspection and maintenance costs.

In recent years, green buildings have developed rapidly with the support and encouragement of many parties. The use of green materials in buildings combined with sustainable operation and maintenance means buildings can play a role in protecting the environment, saving resources, and reducing pollution, which directly affects the construction of ecological cities [52].

The increasing number and scale of green construction projects will undoubtedly become the future development trend of the construction industry [53]. Simultaneously, researchers also pay more attention to the urgent need for the green upgrading of a considerable number of old buildings with high green transformation potential. Sustainable operation and maintenance and transformation are effective ways to improve the performance of existing buildings and achieve low energy consumption and low carbon emissions [54]. Under the premise of improving the comfort of personnel, green operation and maintenance should rely on equipment management, energy management, asset management, and personnel management. The short-term prediction model of public building energy consumption established by the machine learning algorithm [2,55,56,57] is combined with the prediction results to adjust equipment through intelligent operation and maintenance planning and the use of green building materials, allowing for the effective reduction in building energy consumption and the achievement of the green goal.

Besides the material and system control of the building itself, building user behavior, or the ‘human factors’ found in the research of Piper et al. [58], has a significant influence on building performance. Masoso et al. [59] suggested that non-working hours (56%) consume more energy than working hours (44%). Managing the switching behavior of workers related to lights, air conditioners, and other equipment, and determining the correspondence between the equipment and hot and cold climate conditions have a huge impact on energy consumption. In summary, there is great room for the development of the sustainable operation and maintenance of stock buildings, and human factors have a remarkable impact on energy consumption. However, human beings are independent bodies of thought. Various activities are not modeled and will be affected by various conditions. In this study, more events directly and indirectly related to human factors are considered to be added to the operation and maintenance process for improving the effectiveness of the operation and maintenance model.

According to the actual operation status of sustainable building operation and maintenance projects or green building projects, the phenomenon of ‘heavy design and light operation’ is still prominent. The materials and equipment required for the sustainable operation and maintenance of buildings are usually more expensive than for traditional buildings since this part of the price premium often covers additional costs, such as import prices, R&D investment, and more efficient but more expensive building systems [60]. Consequently, more owners are in a wait-and-see state and dare not experience the actual effects of sustainable operation and maintenance. Traditional properties are generally unable to grapple with the relatively complex operation and management needs of green buildings. As a result, the promotion of green operation and maintenance is slow and the actual effect is not as significant as expected.

With the purpose of comprehensively overcoming the above issues, DT technology, which integrates BIM, IoT, intelligent algorithms, and other aspects, is applied to the construction industry. Many researchers have summarized the intelligent operation and maintenance model of DT and applied it to the aerospace industry, manufacturing industry, construction industry, mining industry, and more and more fields after adjusting the model [61,62,63]. With the development of society, the original model can no longer meet new needs, including the exploration of the correlation between factors and results. Besides, the model needs special event factors in different scenarios to mine and realize the self-growth of the model. In this study, a sustainable operation and maintenance model of building infrastructure based on DT is proposed to maximize the value of DT. It involves the analysis of the prediction process, the overall perception and the improvement of energy-saving sustainable operation and maintenance [49], as well as the tracking of building energy consumption changes to further explain how they support daily operation management. The core goal is to establish a sustainable operation and maintenance model for buildings. On the one hand, energy consumption predicts the energy consumption of buildings in order to make buildings sustainable in terms of energy. On the other hand, the model itself can realize iteration, update related factors, and make the model run sustainably.

## 3. Sustainable Operation and Maintenance Process of Building Infrastructures Based on DT

**Figure 2:**
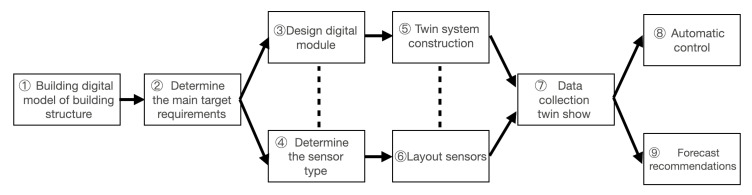
Sustainable operation and maintenance flow chart based on DT.

The digital twin is considered a more comprehensive solution to the needs and research of large public building operations. The digital twin is an intelligent digital system making full use of models and data and integrates artificial intelligence, machine learning, and data analysis. Hence, it can learn and update simulation models from multiple sources and represent and predict the current and future status of physical counterparts. The simulation model is oriented to the product life-cycle process, plays a role as a bridge and link between the physical world and the information world, and provides more real-time, efficient, and intelligent services [35]. At present, there is no unified standard for the practical application process of DT. In this paper, the current application of DT in the construction industry is introduced to sort out the process, as displayed in Figure 2. From the establishment of the basic digital model to the final realization of collecting real-time data and having a dynamic display function, the data set is imported into the algorithm of the system to realize automatic control and feedback adjustment when the data have accumulated to a certain extent and, at the same time, it can predict and make suggestions for future situations [62,64,65].

**Figure 3:**
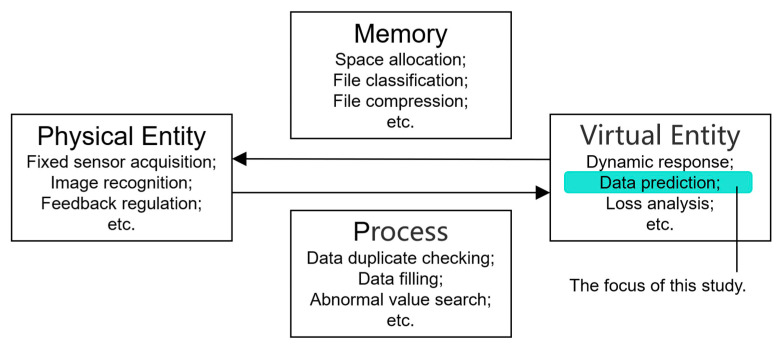
Examples of links involving algorithms in the DT process.

In the sustainable operation and maintenance process combined with digital twin, each link involves a variety of algorithms and computer tools, some of which are summarized in Figure 3. In this paper, the related algorithms of data prediction are studied in depth, and the goal is to further improve the accuracy of prediction.

However, in practical engineering, the DT process only proceeds to the stage of data presentation. The main reasons are described as follows: (a) At present, many large-scale projects are still in the state of independent development. The process involves civil engineering, information technology, interactive design, and other aspects of knowledge content. Therefore, the cross-disciplinary collaborative work of the whole process is one of the key points in the process. Nevertheless, the way to achieve professional cross-integration is different. Thus, the systems cannot be interoperable, the data format is not uniform [66], and DT development costs are high, hindering the comprehensive analysis of larger amounts of data and the achievement of iterative upgrades of similar projects [67]. The standardized use of data can effectively improve the operation efficiency and practicability of the model; (b) Since the data and algorithms used in conventional DT are fixed, the factors concerned in buildings with different functions in the operation and maintenance process are diverse. The model needs to have the ability of self-correlated factor mining and self-growth and to continuously revise the model by expanding factors; (c) The data types and prediction algorithms used in the current DT model are relatively fixed, leading to similar results. Therefore, the benefit for actual operation and maintenance is not significant enough compared with the large amount of capital investment. The algorithm in the model has been developed to a relatively stable state, and the operation accuracy is fixed within a certain range. Under the condition that the algorithm cannot be greatly optimized, this study attempts to refine and disassemble the process of the model combined with the algorithm to realize the innovative application of the algorithm and improve the intelligent operation and maintenance prediction effect.

As suggested above, an intelligent operation and maintenance model with self-growth ability is required. The data analysis process is more intuitive, with the ability to discover abnormal states and explore potential influencing factors. Meanwhile, the model is standardized to improve processing efficiency and enhance the universality effect.

## 4. Establishment of a Sustainable Operation Model for Building Infrastructures

### 4.1. DT Framework of Building Infrastructures

Based on the DT application platform framework, a sustainable DT application platform framework is established according to the development requirements of large-scale building operation and maintenance. The main goal of existing frameworks is efficient operation and safe operation at the functional level. With the emphasis on environmental protection, sustainable operation and maintenance have become a new focus. Therefore, the difference from the current intelligent operation and maintenance system is that the sustainable intelligent operation and maintenance system considers sustainability, efficiency, and security at the functional level. It can not only stress one aspect of the breakthrough but can also coordinate the three aspects to achieve a synchronization upgrade.

**Figure 4:**
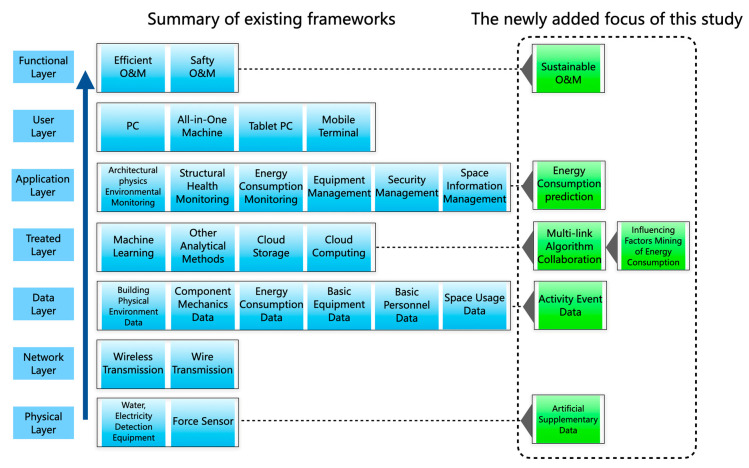
Integration of sustainable operation and maintenance requirements based on traditional operation and maintenance framework.

As demonstrated in Figure 4, the sustainable DT operation and maintenance framework emphasizes the sustainable attributes of each part on the original basis compared with the existing DT operation and maintenance framework. The left side of the figure is a DT operation and maintenance framework integrating multiple articles. The functional layer joins the overall goal of sustainable operation and maintenance. The physical layer enhances the capture of personnel information and adds personnel-related sensors because the program does not set the monitoring data and changes in the activity data the machine system cannot capture still need artificial supplementation. The data layer considers the impact of changing activity time on the results and introduces the activity event data into the framework. The processing layer adopts different algorithms for different links and maximizes efficiency through the collaboration of algorithms. The process of data processing stresses the anomalies between data prediction and results, mines more influencing factors, and continues to supplement the data monitoring range so as to realize energy consumption prediction and auxiliary decision-making at the application layer.

### 4.2. Establishment of DT Model of Building Infrastructures

At present, the research on the energy saving of public buildings mainly combines relatively fixed factors such as equipment and building materials, including enclosure structures, refrigeration, lighting, heating, and elevators [68]. The factors affecting energy consumption also include activity events that are not captured and recorded by sensors. Combining more comprehensive events into the data analysis process can improve the prediction of energy consumption. Especially for large buildings such as commercial operations, being able to combine and master the prediction of events is beneficial to daily business work. The intelligent operation and maintenance of the construction industry are mainly used in functional buildings such as production plants [69,70]. With the continuous improvement of intelligent technology, intelligent operation and maintenance should also pay attention to the needs of commercial operation and maintenance. A six-dimensional model of the sustainable operation and maintenance of large public buildings was established based on the DT five-dimensional model [71]. The overall model can be expressed as:

M_SDTOM-BI_ = (B_PE_, B_VE_, B_SS_, B_DD_, B_CN_, B_SL_) (1)

where B_PE_ represents the physical entity of the target building; B_VE_ denotes the virtual entity of the target building; B_SS_ indicates the whole life-cycle service; B_DD_ signifies the whole life-cycle data; B_CN_ refers to the connection of each component; B_SL_ stands for sustainable links.

B_SL_ is a new part of the model and a key feature of model sustainability. Its connotation includes both the sustainability of the building and the sustainability of the model. Data processing and prediction in the actual operation and maintenance process of building sustainability focus on saving energy and reducing material waste at all times. The sustainability of the model is that the model is not a fixed data source and fixed processing flow model. The model should increase considerations and iterative algorithms without impacting the operation of the original state.

**Figure 5:**
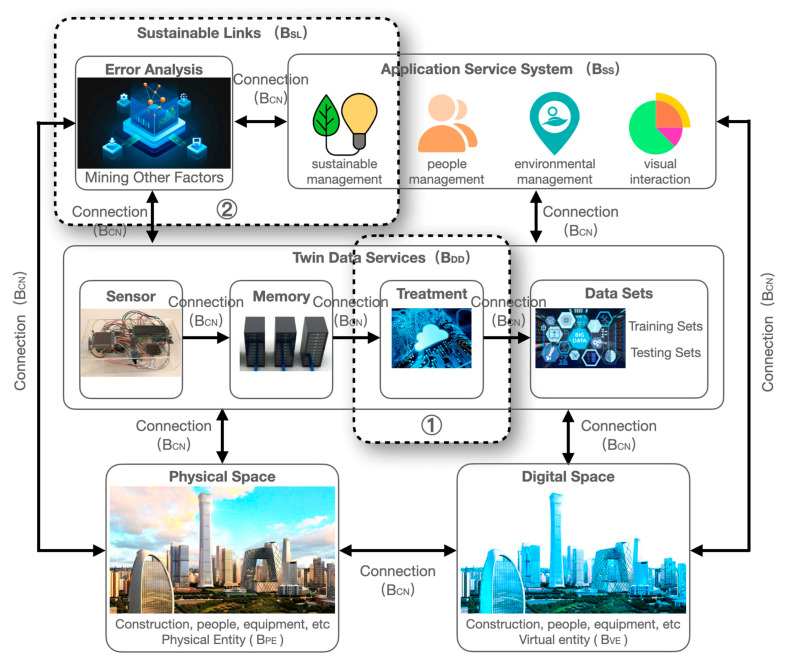
Sustainable DT model of operation and maintenance for building infrastructures (SDTOM-BI).

A sustainable operation and maintenance framework based on DT is proposed according to Equation (1) to facilitate the unified reference of relevant intelligent operation and maintenance concepts. As illustrated in Figure 5, the sustainable dimension is added on the basis of the original model. This is mainly realized by two parts using virtual coils in the figure: ① emphasizing the analysis of energy consumption-related data in the data processing process; ② the application part emphasizes the management of sustainable development. After the application result is obtained, the anomaly analysis step is added. B_SL_ is an important part of the increase in the model. It is the enhancement of the overall model in terms of sustainability. In the application step, it constantly explores the influencing factors, adjusts the weight of the associated elements, and feeds back to the association algorithm of the digital twin system. At the same time, B_SL_ is also an important mining step for errors. In the process of model operation, the application results are optimized and adjusted to feed back better results and adjustment strategies in the physical space. B_SL_ also provides a guarantee for data processing in the process, including the detection of wrong data in the data, the reminder that the sensor is aging or outdated and so on. Based on the above content, it is ensured that SDTOM-BI can complete its own iteration by comparing the results with the actual situation and excavating other related factors to supplement the monitoring and data acquisition process of physical space. In this way, the model can adapt to buildings with different functional characteristics and realize the self-growth and sustainability of the model.

Additionally, there is a difference between the six-dimensional model diagram in Figure 5 and the arrow pointing of the DT five-dimensional model diagram. This diagram is directed in one direction following the application process. The basic model data of the physical space are directly applied to the digital space. The data obtained by the sensor are applied to the digital space after data processing, allowing the digital space to form a DT model. Various types of simulation and management work are performed based on the DT model. With the results in the application process, the abnormal situation is discovered, and the related factors are added to the information collection of the physical space so as to form a closed-loop sustainable DT operation and maintenance model for large public buildings [49,72,73,74].

**Figure 6:**
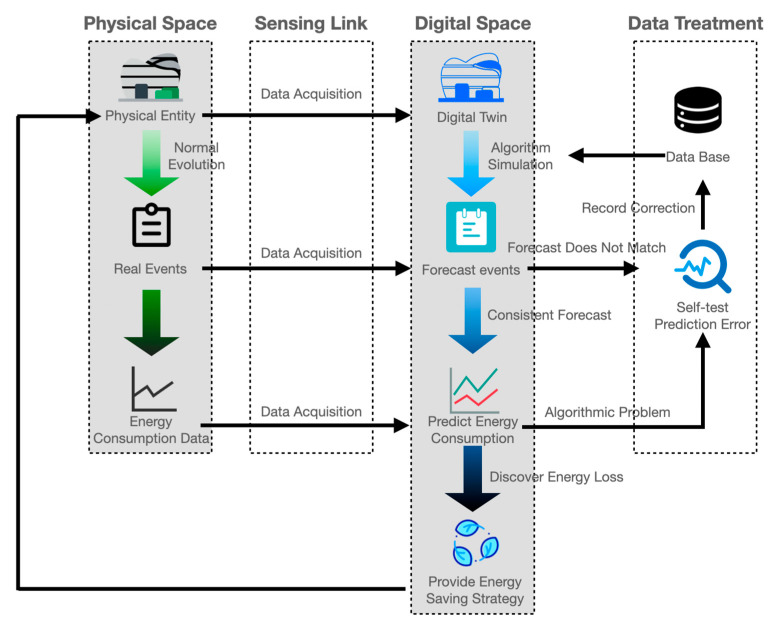
The DT process model of gymnasium sustainable operation and maintenance.

The model is data-driven, and the process of data-based analysis is the main research part of the current intelligent operation and maintenance process [74]. Figure 6 demonstrates a sustainable DT operation model of large public buildings drawn with data as a logical relationship, which is detailed below.

The sustainable operation and maintenance model of this study is a universal model, and different types of buildings can be substituted into this model. Nonetheless, the necessity and effect need to be considered. Large buildings have large volumes and regional divisions in the same room only for lighting, and these are not available in small buildings. This model requires the supporting IoT data collection equipment. In other words, the use of this operation model in small buildings may not necessarily have energy-saving effects, while wasting resources. Other types of large public buildings can apply this model in combination with the scene.

**Figure 7:**
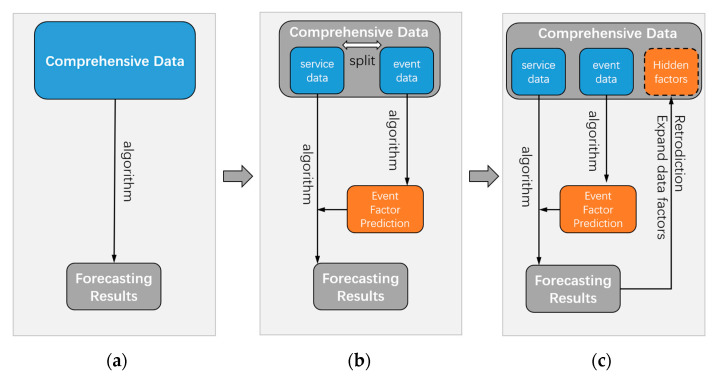
The data supplementation and correction of the green operation and maintenance DT model. (**a**) Regular process. (**b**) Add event factor prediction phase. (**c**) Reverse tracing of hidden factors.

Compared with the conventional energy-saving operation and maintenance model, the biggest difference is that in Figure 7a the data are generally collected comprehensively by the sensor, and the coupling relationship between each datum and the target prediction result is obtained by the algorithm. However, the operation and maintenance process in reality is not all associated with the monitoring data and the prediction results, and a considerable number of unknown factors are not monitored or cannot be monitored. The data part in this model is exhibited in Figure 7b, which is divided into operation data and event data. The operation data are the standard data that can be monitored by the sensor on a daily basis, and the event data are the occurrence of events that cannot be recorded by the sensor on a standardized basis, such as sudden disasters, temporary construction, and a company coming to the gym to organize competitions. The event data are used to first predict event factors, so as to supplement and correct the operation data to predict the results. In the further application of the operation and maintenance model, as demonstrated in Figure 7c, if there is a large deviation in the prediction results, the uncertain event factors can be backtracked and mined, and the potential events can be queried to continuously revise the model.

Therefore, the DT operation and maintenance model summarized in this study is no longer the traditional collection of fixed data and export results but an evolutionary model that can continuously supplement itself and iterate and upgrade.

### 4.3. Digital Twinning and Visualization for Building Infrastructure

#### 4.3.1. DT Data Acquisition Method

Data are the foundation of all intelligent operations. The current data acquisition process is relatively fixed. In the existing data-driven building energy consumption analysis research, the data sources can be divided into two main categories: measurement data and simulation data [23]. Measurement data can be directly collected from building automation systems (BASs), energy meters, weather stations, field surveys, and IoT sensors. Measurement data can reveal the actual operation of buildings and their energy systems. In recent years, the rapid development of IoT-related technologies has contributed to simpler and cheaper data collection. They are widely used in smart life [75] and remarkably improve device stability [76]. However, sensor-based data collection methods still need to strictly check and verify data quality, which is the main task of data cleaning. Simulation data are collected from physics-based models and simulation tools. Commonly used simulation tools, including DeST [77,78], TRNSYS [79], EnergyPlus [80,81,82], Ecotect [83], and eQuest [81], adopt data-driven methods for the simulation of predetermined conditions. However, the simulation data from previous studies mainly pertain to commonly occurring situations, and fail to capture the dynamic interactions between the abnormal triggering events and energy consumption outcomes. Since one of the main contributions of the proposed approach is to the sustainable operation model in this study, one of the key functions is the backtracking and mining of abnormal events under the influence of multiple events. The purpose is to enable the model to continuously discover new correlation factors and extend the correlation factors of the model. Therefore, multi-source measurement data (e.g., environmental data, weather data, activity data, equipment data, etc.) are implemented in this study.

#### 4.3.2. DT Data Processing

Maintaining data integrity, effectiveness, and interoperability is one of the crucial issues for intelligent operations. Before analyzing data, the data should be filtered and organized to avoid interference from erroneous data [84]. In the process of building green operation and maintenance, the following data format framework is designed under the consideration of the characteristics of the model and the collection process of the measured data of the IoT. According to this framework, the data types can be continuously expanded, and the unnecessary workload can be reduced in the follow-up research. The data framework is divided into five parts: environment, events, equipment, energy consumption, and personnel factors, as described in Table 1.

#### 4.3.3. DT Data Visualization

In addition to the twinning of data, visualization is critical in digital models. The data transmission format can be standardized by making a preform in Unity3D, forming a modular application. This can assist in improving the construction efficiency of DT applications and the ease of use of applications.

**Figure 8:**
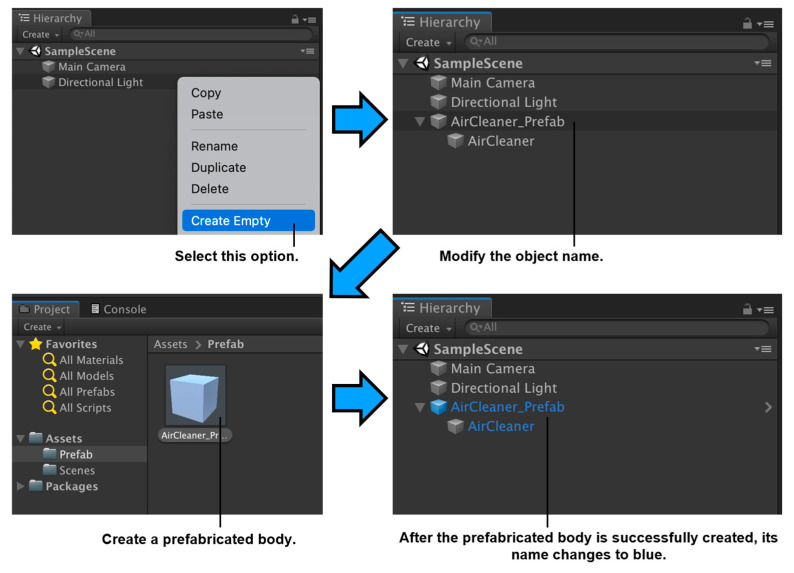
Prefab creation process in Unity3D.

The duplicate objects present in the building scenario, such as the same lights, windows, doors, and air conditioning, are set to the Prefab component in the system. The visual layout of the scene objects can be quickly completed by dragging the Prefab component. Figure 8 displays the operation flow in the software.

**Figure 9:**
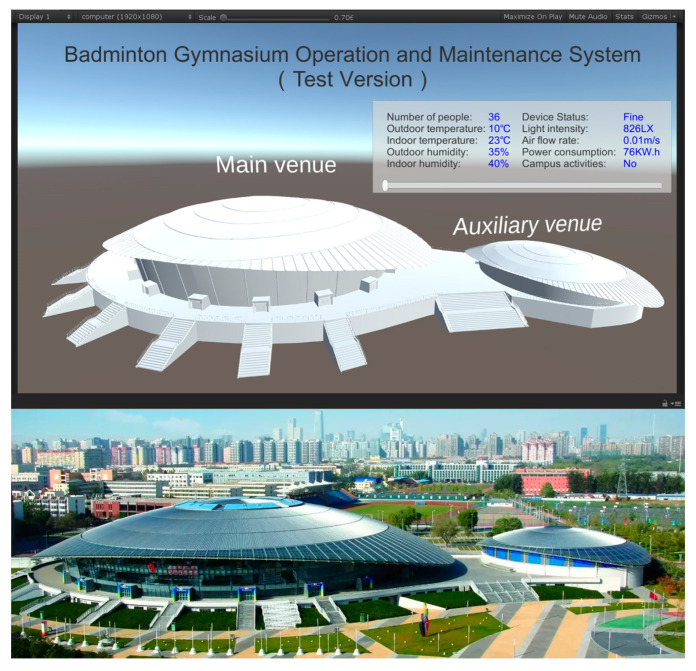
Model sketch of the gymnasium.

According to the above process, the model matching the target building can be quickly obtained, as shown in Figure 9. This research focuses on the prediction process. The model is only used as a carrier to cooperate with the test, so the structural model is made without deep rendering.

Following the creation process of Prefab, we can set the size of objects and code scripts into Prefab according to our needs. Therefore, the collected data and early warning can be visually presented in the model, and a complete DT system is established, including data collection, data processing, data forecast, and data presentation.

### 4.4. Event Prediction

Regarding the prediction of events, the daily operation and maintenance method indicates the occurrence of events in the same period of the previous year. This method is a static analogy, not a dynamic prediction. Machine learning can combine the analysis of data sets to form predictions of future data, while such predictions need to be based on comprehensive data, suggesting that all influencing factors can be recorded and expressed with data. Generally, there are no such comprehensive data for the operation and maintenance of gymnasiums. Meanwhile, social activities, sports competitions, and other event factors will exert significant influences on visitors. Hence, the prediction step of events is added before the energy consumption prediction step in this prediction model. Changes in different conditions during the operation and maintenance process may induce changes in the results and have an impact on the prediction of events. For basic events, the Bayesian formula is adopted to calculate the probability [85,86,87]. When the event of a step is affected by multiple conditions and each event is independent, it is expressed as follows according to the Bayesian formula:

(2) $$P(X_{i}|Y_{i})=\frac{P(X_{i})P(Y_{i}|X_{i})}{\sum_{j=1}^{n}P(A_{j})P(Y|X_{i})}$$

(3) $$=\frac{P(X_{i})P(Y|X_{i})}{P(X_{1})P(Y|X_{1})+P(X_{2})P(Y|X_{2})+\ldots +P(X_{n})P(Y|X_{n})}$$

Researchers can predict the time or infer the occurrence of conditions according to the probability.

The prediction part of the model combines various data collected in the target physical space X = { X_1_, X_2_, …, X_n_}, following the chain rule of the Bayesian network:

P(X_1_,X_2_,…,X_n_) = p(X_1_)p(X_2_|X_1_)p(X_3_|X_1_,X_2_)… p(X_n_|X_1_,…X_n−1_) (4)

(5) $$=\prod_{i=1}^{n}p(X_{1}|X_{2},\ldots X_{i-1})$$

Under the known causal relationship, the chain rule can be constantly simplified to obtain the probability of the event. The above method is employed to predict the use of equipment and sites. From another perspective, whether it is required to overhaul equipment or other conditions affecting operation and maintenance can be determined according to the comparison of probability with previous data.

### 4.5. Energy Consumption Prediction

After judging the events without data through the probability and Bayesian network model, the building energy consumption is predicted by the machine learning algorithm with the known data. Zeki-Suac et al. [88] utilized artificial neural networks (ANN), decision tree, and random forest (RF) machine learning algorithms to model the energy consumption of public buildings, respectively. The results demonstrate that the random forest algorithm has higher prediction accuracy in prediction modeling [89].

There are many factors related to the energy-saving operation and maintenance of large public buildings. This paper takes the study of large stadiums as an example. According to the literature and actual interviews with many staff in the stadiums, lighting equipment, temperature control equipment, and air conditioning equipment are directly connected with the changes in energy consumption in stadiums. The use of these pieces of equipment is affected by various factors, such as weather conditions, the impact of social activities, and holidays.

**Figure 10:**
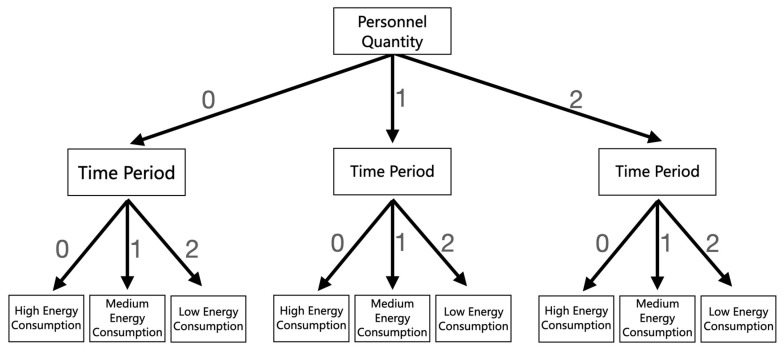
Establishment of the decision tree model.

The classification region tree (CART) is the foundation of random forest (RF). According to the characteristics of building energy consumption data, the decision tree model between factors and energy consumption can be established by using a cart algorithm [90]. Figure 10 is taken as an example to introduce the establishment method of the decision tree model. First, the weather is divided into three types: “0” (sunny days), “1” (cloudy days), and “2” (rainy days). Time periods are divided into three types: “0” (morning), “1” (afternoon), and “2” (night). According to the above types of branches, a decision tree model can be established, and different situations correspond to high, medium, and low energy consumption values, respectively.

In the split of each node, only m randomly selected features instead of all prediction variables (random feature selection) are considered. The creation of decision trees is repeated m times. This study selects m decision trees to form a randomly generated “forest”. For each tree, the same number of *n* samples are randomly selected by bootstrap resampling to form a new training set. The unselected samples are called out of bag (OOB). Random forest overcomes the instability of a cart by using a group of trees to replace the prediction of a single tree. Generally, the training set and test set are established by random sampling with the return to avoid the influence of extreme conditions. In this way, the model effect is significantly improved concerning accuracy and stability.

The repeated sampling of the original data set generates each regression tree in the RF. About one-third of the samples were not extracted during each repeated sampling, which formed a control data set [91]. The establishment of a random forest mainly includes four points: (1) bootstrap resampling, (2) random feature selection, (3) full-depth decision tree growing, and (4) out-of-bag (OOB) error estimate. The random forest can be utilized to verify the error with out-of-package (OOP) data in the process of building a forest. This verification is an unbiased test in most cases [92].

**Figure 11:**
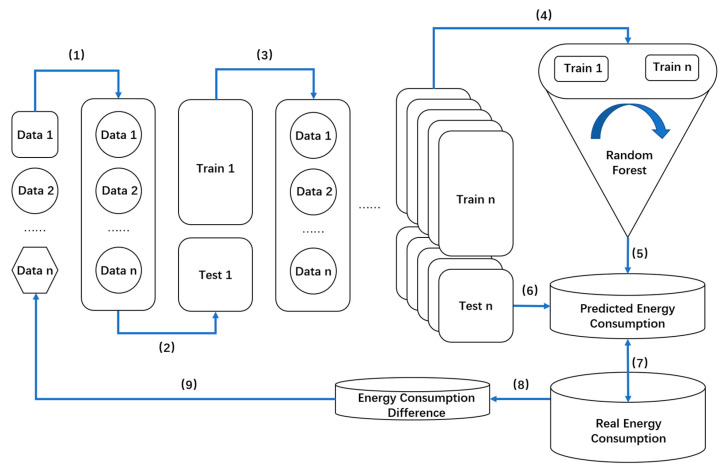
Random forest energy consumption prediction model.

In the application of this sustainable operation and maintenance model, the random forest prediction of energy consumption on multiple factors is conducted based on the prediction of events in 4.2.1. The steps are detailed in Figure 11: (1) collecting multidimensional data; (2) data cleaning (data are summarized in different formats and scales into the same data set); (3) data segmentation (data are randomly selected to form a training set, the training set data account for 50–70% of the total data, and the rest are used as the test set); (4) putting the extracted data back into the total population, and repeating the previous step many times to increase the number of test sets and training sets; (5) training the model (the training set and test set are introduced into the random forest model for training, and the number of spanning trees is adjusted, so as to make the accuracy of random forest prediction stable); (6) realizing the prediction, and adopting the trained model to predict the energy consumption; (7) by adjusting the predicted influencing factors and comparing the changes in energy consumption, it can be concluded that energy consumption can be reduced while maintaining comfort and building functionality; (8) comparing the actual energy consumption and the predicted energy consumption to reveal the abnormal energy consumption difference data items; (9) reversing the possible abnormal influencing factors, paying attention to the impact of equipment safety and social activities on operation and maintenance, and assisting personnel management [90,93].

The purpose of this study is not to improve prediction accuracy, because in fact the accuracy of the current mainstream prediction methods is sufficient for daily operation and maintenance. Based on the existing algorithms and digital twin framework, this paper increases the backtracking of events while maintaining the prediction ability of the algorithm. The biggest difference between this and other algorithms is that in the data cleaning process the outliers will not be eliminated or replaced but remain in the overall data. These outliers also correspond to a certain state of the operation and maintenance process, only because of the influence of some factors. The data are different from the usual situation. Therefore, what this paper does is to retain the complete data, combine the algorithm to maintain predictive ability, and increase event backtracking ability.

## 5. Model Application Verification

### 5.1. Background of the Experiment

The DT model is combined with the badminton venues of the Beijing Olympic Games to verify the application with a competition venue and a room as an example. The foundation of intelligent operation and maintenance is data. There are significant differences in the data generated by different types of buildings [94]. Compared with other large public buildings, the continuity of large stadiums and gymnasiums is that they occupy large spaces and have complex personnel. The difference is that the gymnasiums have higher requirements for competition venues. The Olympic Badminton Stadium targeted by this experiment has strict requirements for light, temperature, humidity, and indoor wind speed. Moreover, the instantaneous flow of people entering and leaving the stadium is huge. In the daily operation process during a non-competition period, social events have a considerable impact on visitors to the venue.

First, the DT relationship is established to realize a dynamic real-time response, and then the data obtained by the DT are analyzed to realize the result feedback. The design content of the experiment is shown in Table 2.

### 5.2. Data Collection

The lobby environment of the badminton stadium is simulated and tested. A public space is selected as the test site to simulate and record the data. The electricity consumption data are obtained from the Green Building Technology Center of Beijing University of Technology. The external environment data of the building are obtained through the meteorological bureau and various websites. Based on the Arduino hardware platform, a response sensor is developed and manufactured to obtain data such as personnel and environment in the space. The development version adopts Arduino uno R3, which has high-cost performance and rich relevant library files. Environment-related sensors are selected to collect the relevant data from Olympic badminton venues. The accuracy is sufficient for model verification. The specific equipment models and functions are described in Table 3.

**Figure 12:**
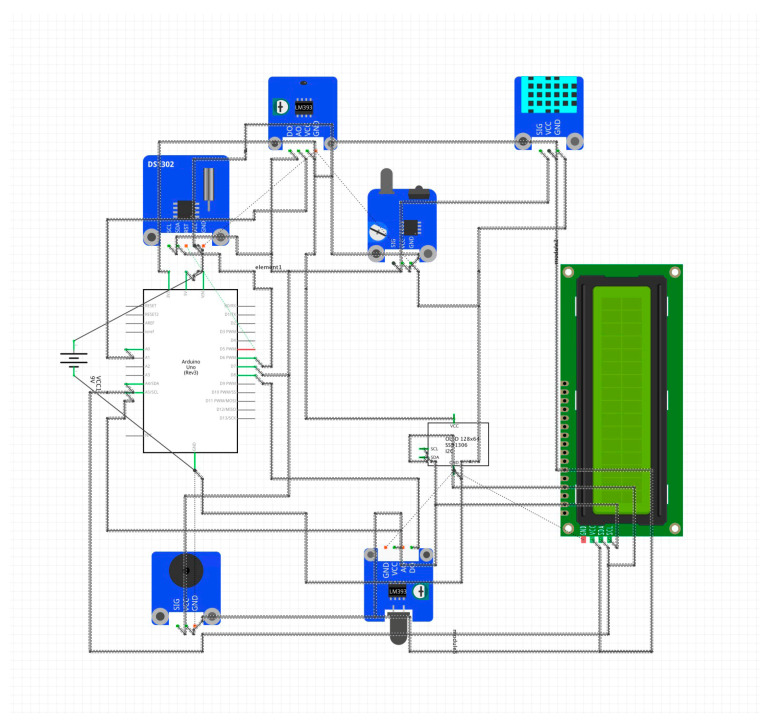
Sensor connection diagram.

**Figure 13:**
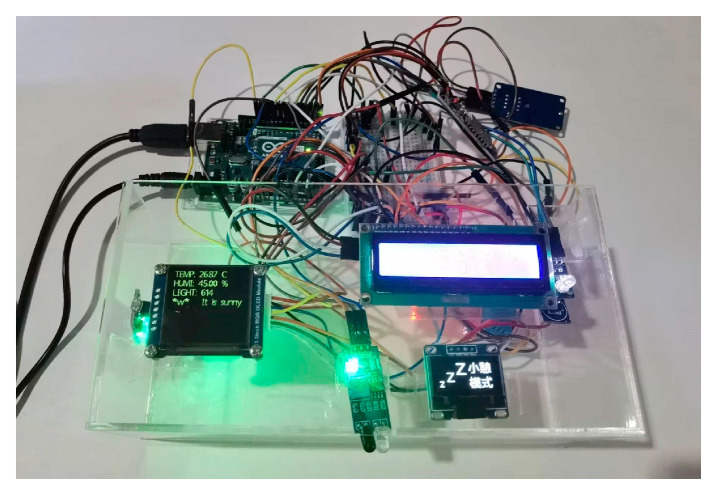
Data collection box-type device.

**Figure 14:**
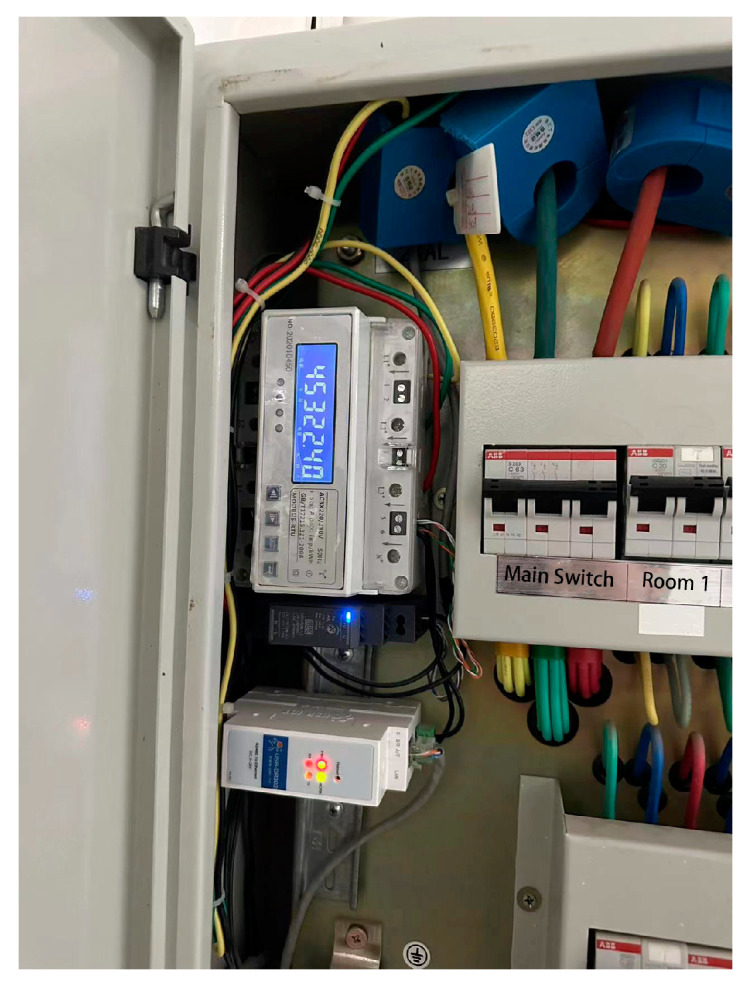
Multifunctional electricity meter is installed in the electric box.

**Figure 15:**
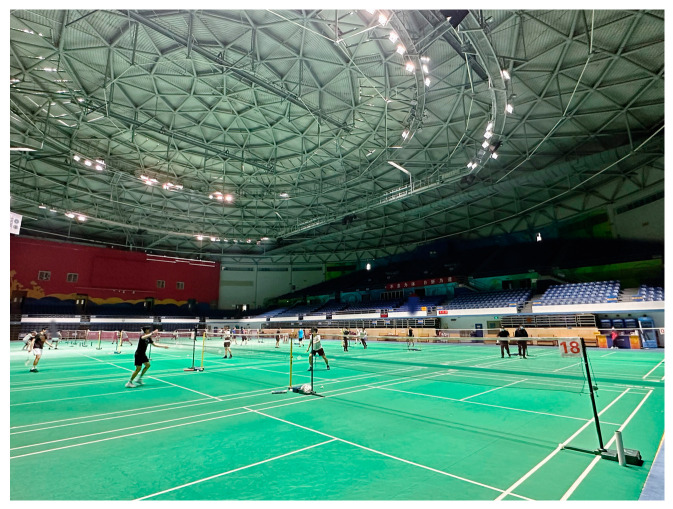
Monitoring of activity images within the venue.

As shown in Figure 12, connecting sensors related to Internet of Things devices can continuously expand sensor types, create data acquisition box devices, and connect Wi-Fi for real-time data transmission. As shown in Figure 13, a small detection device box monitors data in local areas. The data collection device is equipped with a miniSD card for local storage, which circularly writes data as backup data to avoid network anomalies. As shown in Figure 14, a multi-function meter is connected to each electric box to obtain the power consumption of each circuit. As shown in Figure 15, the indoor space of the experimental target building collects and records personnel activity status.

### 5.3. Data Sorting and Analysis

#### 5.3.1. Data Collation

The energy consumption is sorted according to the data recorded by the comprehensive power consumption sensor. The power recording is performed every second, and the data time is from 11:47 a.m. on 12 January 2022 to 9:46 a.m. on 8 June 2022.

**Figure 16:**
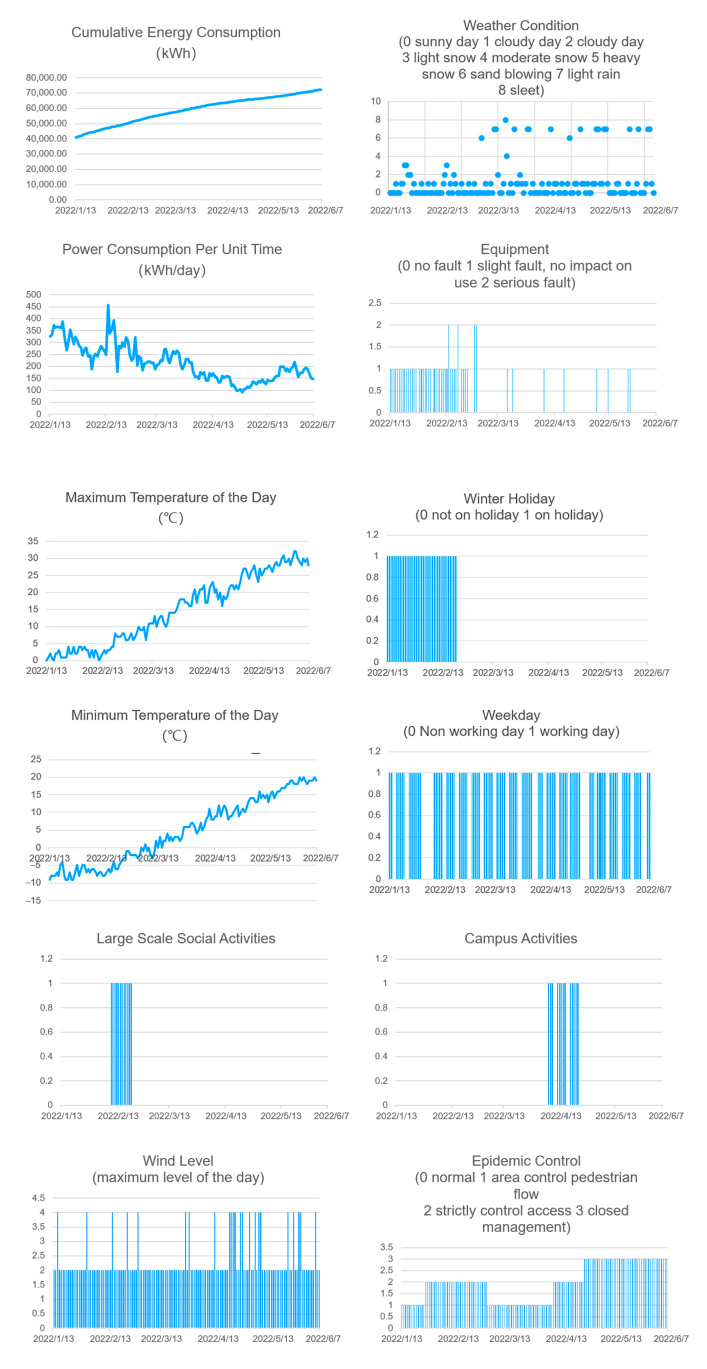
Preliminary analysis of various data.

In the process of data processing, the daily maximum and minimum temperatures, weather conditions, and wind speeds can be obtained through the weather data of the National Meteorological Administration; social activities related to campus production are searched and recorded in combination with public news; relevant activities in the school are obtained through the campus network; epidemic prevention and control are involved in the experimental data recording period, exerting an impact on the use of the site. Therefore, epidemic control is also included in the datasheet as a separate item. The time scales of various data records are different. The time interval of the data is unified with the day as the unit, and the power consumption is converted into the energy consumption per unit of time (day). As the power consumption data are not complete on the first day and the last day of the data, they are omitted. The final data set is from 13 January 2022 to 7 June 2022, with a total of 146 natural days. Figure 16 shows the change of data.

#### 5.3.2. Preliminary Data Analysis

The preliminary mapping analysis is conducted on a single datum to observe the trend change and screen the wrong value.

As revealed from the chart, all data have significant change trends and different trends. It is suitable to find the coupling relationship behind them. The cumulative energy consumption value, power consumption per unit of time, maximum temperature of the day, and minimum temperature of the day are represented by a broken line chart. The fluctuation trend is observed. Equipment conditions, holidays, working days, wind levels, large-scale activities related to society, school activities, and epidemic control are illustrated with a two-dimensional histogram. The weather conditions are divided into eight kinds of data, and the situation is more complex, characterized by a scatter diagram.

This data includes 3 weeks of strict COVID-19 control, 2–3 weeks of ‘large-scale social activities’, and 4 weeks of winter holidays. If it is a routine data cleaning process, these data will be removed as abnormal data and only stable operating conditions are considered. However, this is inconsistent with the actual situation. In the actual operation and maintenance process of the campus badminton venue, the beginning of the winter vacation is directly related to the opening status of the venue equipment and the number of staff. In recent years, the control of the epidemic situation has also been directly related to the development status of the activities, so these data cannot be removed and ignored. In this project, all data retention is brought into the model in order to train and validate predictive models that are closer to the true operational state.

#### 5.3.3. Event Prediction Application

The data of the event step are obtained by interviewing the staff on duty. The probability that the equipment in a certain court of the badminton hall is in good condition is 0.95. The probability that someone will play and use the court is 0.88 when the equipment is in good condition. The probability of using the court is 0.25 when the equipment has issues, including insufficient lighting. Without the consideration of the impact of other events, the probability of good startup of the site equipment when no one uses this site one day is also calculated.

That is, A = {use the site} B = {good equipment}

(6) $$P(\bar{A}|B)=0.12,\;P(\bar{A}|\bar{B})=0.75,P(B)=0.98$$

From the Bayesian formula:

(7) $$P(B|\bar{A})=\frac{P(\bar{A}|B)P(B)}{P(\bar{A}|B)P(B)+P(\bar{A}|\bar{B})P(\bar{B})}=\frac{0.12\times 0.98}{0.12\times 0.98+0.75\times 0.02}=0.88$$

The result is significantly lower than the good probability of daily equipment. The automatic system can provide an equipment inspection strategy in accordance with the dynamic change of probability. The above methods can be predicted in the basic step, which is limited to the case that each condition is independent. Nevertheless, it is more affected by multiple conditions.

The use status of badminton venues is still taken as an example. During the routine operation and maintenance of venues, other impact conditions influencing the use of venues are expanded. Table 4 lists the probability of playing under different conditions and the probability of using venue 1.

Since all conditions are interrelated, each condition is represented by a number as $X_{i}$, i = 1, 2, 3, 4, 5, 6. The chain rule of the Bayesian theorem should be employed to express the structure of joint probability distribution. In a comprehensive case, the probability of playing is expressed as:

(8) $$P(X_{1},\;X_{2},\;X_{3},\;X_{4},\;X_{5},\;X_{6})$$

(9) $$=P(X_{1})P(X_{2}|X_{1})P(X_{3}|X_{1},\;X_{2})P(X_{4}|X_{1},\;X_{2},\;X_{3})P(X_{5}|X_{1},\;X_{2},\;X_{3},\;X_{4})\;P(X_{6}|X_{1},\;X_{2},\;X_{3},\;X_{4},\;X_{5})$$

The model is established according to the Bayesian network, as illustrated in Figure 13.

**Figure 17:**
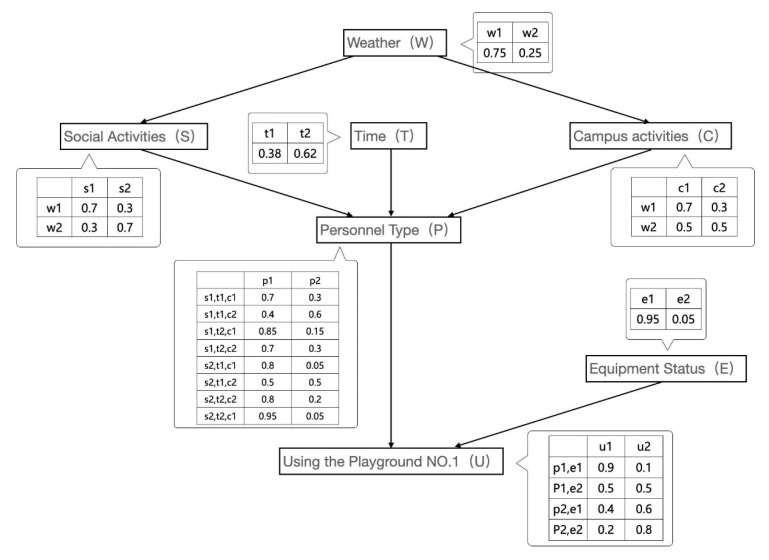
Probability distribution based on Bayesian network.

Figure 17 contains six conditions and one result, respectively:
Personnel Type (P)p1 Studentp2 Social personnelWeather (W)w1 Fine weatherw2 Bad weatherSocial Activities (S)s1 Actives2 No activityCampus activities (C)c1 Campus activitiesc2 No campus activitiesTime (T)t1 Non-working hourst2 Working hoursEquipment Status (E)e1 Good equipmente2 Equipment abnormalityUsing the Playground NO.1 (U)u1 Useu2 Not in use

According to the Bayesian network diagram, the joint probability distribution can be expressed as:

P(W,S,T,C,P,E,U) = P(W)P(T)P(S|W)P(C|W)P(P|S,T,C)P(E)P(U|P,E) (10)

When the outdoor weather is good, no badminton-related social activities are held, campus badminton activities are held, and the badminton stadium is well equipped. The joint probability of students using venue 1 during working hours is expressed as:

(11) $$\begin{array}{l} \;P(w1,s2,t2,p1,e1,u1)\\ =P(w1)P(t2)P(s2|w1)P(c1|w1)P(p1|s2,t2,c1)P(e1)P(u1|p1,e1)\\ =0.75\times 0.62\times 0.3\times 0.7\times 0.95\times 0.95\times 0.9\\ =0.08 \end{array}$$

## 6. Verification and Discussion

Combined with the previous content to carry out specific calculations and verification, Python language is used to write the algorithm. The date column is removed from the data and stored as a CSV format data file. The encoding format is GBK. The total sample size is 146. Pycharm is adopted to execute the algorithm program, adjust the training set, and test set for three sets of validation tests.

### 6.1. Three Groups of Tests

1. Classify the energy consumption according to the monthly average energy consumption level and use random forest to sort the weight of influencing factors.

Under the principle of non-replacement extraction, 25 items of data were extracted every month from February to May, and 10 items of data were extracted in January. A total of 110 items of data accounted for 75.34% of the total data set. Then, the test set data were randomly selected from the data of the total sample volume of 146 items of data based on the non-return extraction principle. The random data should involve each month, and a total of 24 items were extracted, accounting for 16.44% of the total data.

**Figure 18:**
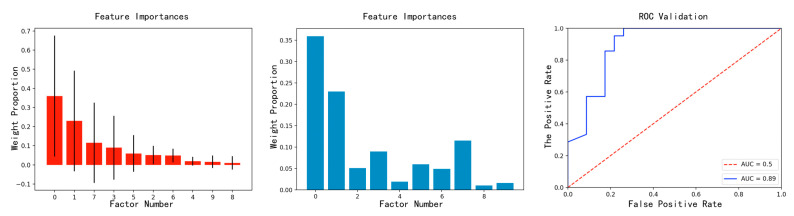
Analysis of influencing factors.

Table 5 shows the weight ranking obtained by substituting the training set into the algorithm. As shown in Figure 18, a bar chart for weight comparison can visually show the weight ratio. The value of the area under the curve (AUC) of the receiver operating characteristic (ROC) is 0.89, indicating that the model is reliable.

2. Bring the specific value of energy consumption into the random forest algorithm for result operation:

The average absolute error is 18.717127755102066;

The call model run time is 0.010010004043579102;

The influence weight values of all features are shown in Table 6.

**Figure 19:**
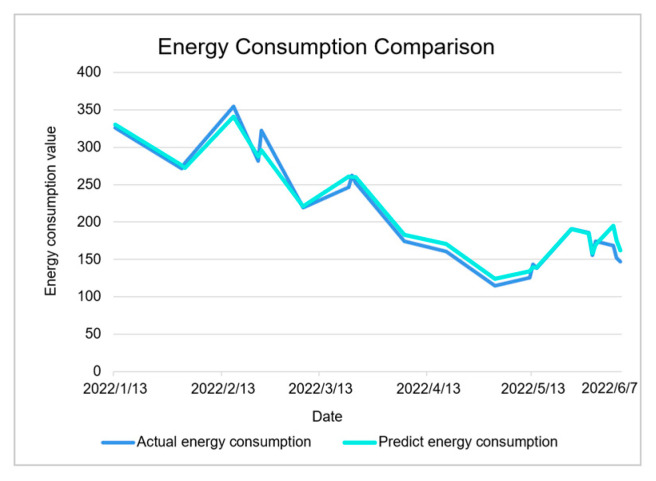
Energy consumption comparison line chart.

**Figure 20:**
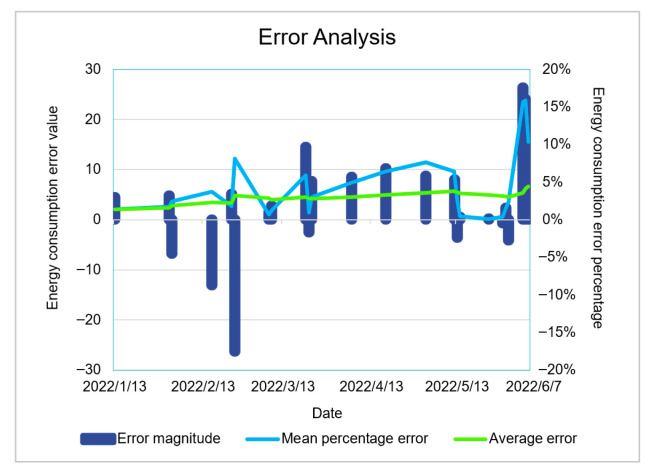
Energy consumption error analysis diagram.

As shown in Figure 19, the prediction results are consistent with the trend. As shown in Figure 20, the error value of the prediction results represented by the histogram, the light-blue broken line is the percentage of the absolute value of the error, and the maximum absolute value of the error appears on June 5, which is 15.86%. This is because there are no training data available for the previous 6 months. The minimum absolute percentage error is 0.38%, which appears on May 29. The light-green broken line is the change of the average percentage of absolute error. The overall average is 4.4%, indicating that the average prediction accuracy is 95.6%. The prediction is highly reliable and the model is available.

3. Detect abnormal states using the prediction results of random forest energy consumption.

The data training set and the test set are regenerated. If there is no return of the 146 pieces of data in the total sample volume, 25 pieces of data are extracted as the test set. These 25 pieces of data are composed of 4 pieces of data with serious equipment abnormalities and random data each month. The remaining 121 pieces of data of the total sample are adopted as training sets to train the random forest model.

The prediction and comparison results are shown in the following figure.

**Figure 21:**
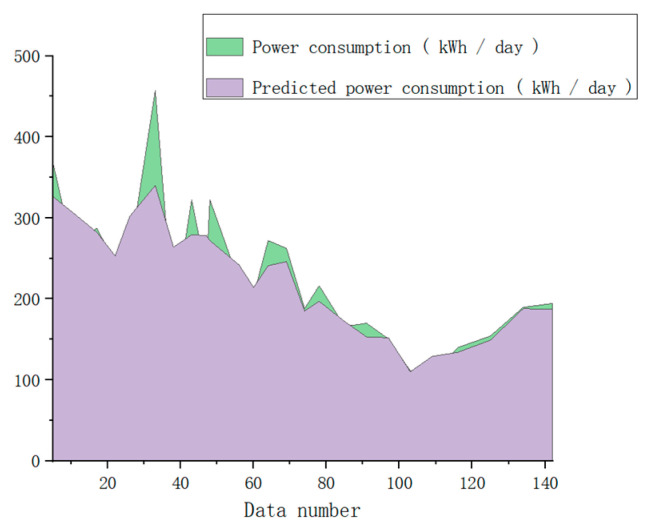
Energy consumption trend chart.

**Figure 22:**
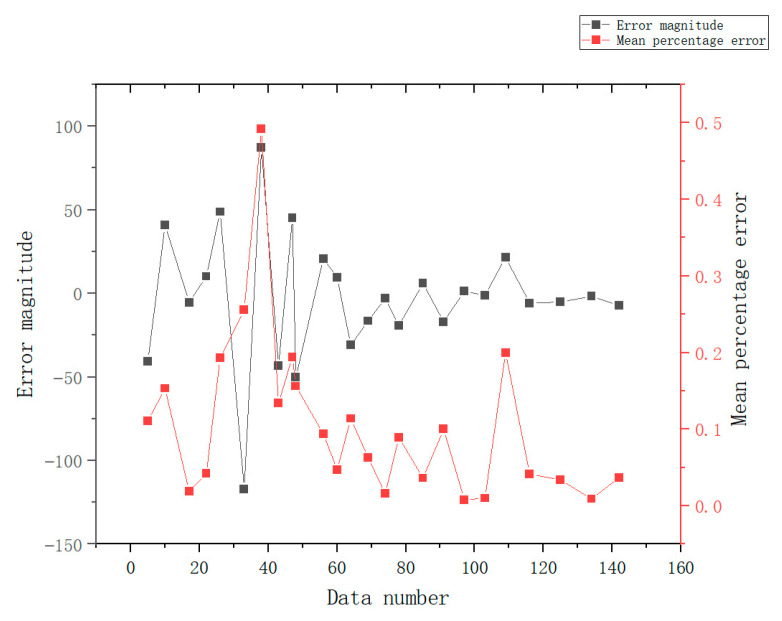
Error value line chart.

As shown in Figure 21, it can be clearly seen that the energy consumption comparison has an error value, and the size of the specific experimental data error value is shown in Figure 22, where the average error percentage indicates the prediction result with an error greater than 15%. The corresponding date is recorded as the date of the abnormal data. According to the data abnormal days to check the corresponding date, the results are shown in Table 7.

It can be observed that among the seven abnormal values obtained from this model, there are four dates when all equipment is seriously abnormal, one date of a snow day and non-working day, one date within the first week of strict epidemic control, and one date with an unknown reason. This test verifies that the model can perform feedback queries of influencing factors according to the results. In addition, the probability of equipment abnormality can be obtained through the results through the time prediction step of the Bayesian network. Simultaneously, it can judge whether there are other influencing factors according to the results and assist operation and maintenance management personnel in discovering unknown risks and operation opportunities.

### 6.2. Discussion and Suggestions

Based on the above process and results, SDTOM-BI and targeted calculation methods can effectively predict energy consumption changes and accurately find the date of abnormal states, and the results are satisfactory. Combined with the results of the experiment, the following suggestions are given for the sustainable operation and maintenance of buildings:

(1) In the aspect of building energy conservation, the start-stop adjustment of equipment should be carried out in combination with possible activity events to avoid unnecessary energy waste.
(2) In the process of building operation and maintenance, the automatic inspection of equipment through computers and other equipment can quickly and accurately query the abnormal state, assist the operation and maintenance personnel in finding the specific abnormal reasons in time, and should be more comprehensively promoted and enabled.
(3) We can make full use of the computer’s ability to process data to conduct reverse queries on the influencing factors of operation and maintenance. Even if the influencing factors of the operation and maintenance process are adjusted, the auxiliary operation and maintenance personnel can better carry out the operation and maintenance work in combination with the latest influencing factors to make the operation and maintenance system sustainable.

The effective scope of this research is aimed at large-scale infrastructure, which is characterized by large volume, high energy consumption, multiple functions, and relatively single owners. Such a building operation and maintenance process has strong regularity data that can be mined. Small civil buildings are not recommended for use. Due to the large number of owners, the space use and equipment use in the building are extremely free and unified operation and maintenance management cannot be carried out.

## 7. Conclusions

Building infrastructures have a significant energy consumption in daily operation, yet the operation and maintenance processes of building infrastructures are not properly monitored, leading to limited strategies. Therefore, a sustainable digital twin model of operation and maintenance for building infrastructures (SDTOM-BI) is proposed in this paper. This proposed model is illustrated and verified via a large-scale gymnasium as an example. Through the mining of the three-part progressive relationship of ‘factor-event-energy consumption’, the model has the ability to backtrack and increase the model-related factors and establish a framework and model suitable for the sustainable operation and maintenance of building infrastructures. The main feature of the proposed method is the combination of the digital twin operation and maintenance model to strengthen the sustainable link so that the model can continuously mine and update the influencing factors of operation and maintenance and can iterate itself, realize the sustainability of the building and the operation and maintenance model itself, and form a good cycle. The conclusions of this study are drawn as follows:

(1) By considering the structural attributes, functional attributes, environmental factors, event factors, and energy consumption characteristics of building infrastructures, combined with the digital twin model, Bayesian network and random forest algorithm, the framework makes the correlation factors between data clear.
(2) The DT prediction model is divided into two parts: event prediction and energy consumption prediction. Therefore, the model can continuously reveal the influencing factors. Optimizing the prediction performance through experimental verification, combined with 146 days of measured data to verify, the model AUC of ROC is 0.89, indicating that the model is reliable. In the energy consumption prediction stage, the minimum absolute error percentage is 0.38%, and the average accuracy rate is 95.6%, predicting good results. During the exception event tracking phase, all six hidden test exception data were discovered; the model has excellent event mining capabilities.
(3) Since the prediction of events is added to the model, the model can reverse the known energy consumption to deduce which events may occur and assist the daily operation or safety monitoring of the stadium. The ability of event backtracking and factor expansion makes the model an evolutionary model that can be iterated and upgraded.

The above research results reveal that the sustainable operation and maintenance model of building infrastructures based on DT can establish an intelligent operation and maintenance system, standardize data acquisition, and provide automatic energy-saving strategies through algorithms. The DT model combines the returned data with 3D real-time rendering technology to achieve a more accurate dynamic DT.

This operation and maintenance model still has certain limitations. For large-scale public infrastructure buildings, it is characterized by large numbers of questions, single owners, high energy consumption, and complex related factors. It is not suitable for small civil buildings. Because of its small number of single questions and many owners, it is inconvenient to obtain and manage the centralized data, and the indoor environment is highly personalized.

In the future, we can also deepen the data in a more comprehensive and accurate way. The digital twin model combines the returned data with 3D real-time rendering technology to achieve more accurate dynamic digital twins. The specific research directions include the following:

(1) There could be more types of event factors and operation and maintenance processes of energy consumption, benefits and other factors associated with the relationship between mining.
(2) The method of event data collection could further expand and improve the level of automatic voice.
(3) Digital twin systems could better predict the results of simulation display and improve the practical application effect.

## Figures and Tables

**Table 1:** Building infrastructures data format framework.

Environment			Equipment		
Maximum air temperature	Tem_H	float (preserves 1 decimal place)	Air conditioning status	AirCon_1 AirCon_2 ……	int (0 start no fault, 1 start minor fault, does not affect use, 2 start and serious fault, 3 not start)
Minimum air temperature	Tem_L	float (preserves 1 decimal place)	Light status	Light_1 Light_2 ……	
Wind level	Wind	int	Heating status	HeatEqu_1 HeatEqu_2 ……	
Weather types	Weather	int (0 sunny, 1 cloudy, 2 cloudy, 3 light snow, 4 medium snow, 5 heavy snow, 6 blowing sand, 7 light rain, 8 sleet)			
**Event**			**Energy Consumption**		
Medical prevention and control	Prevent	int (0 normal, 1 area control pedestrian flow, 2 strict control of access, 3 closed management)	Electrical energy (kW·h)	Elec	float (preserves 1 decimal place)
Social activities	Act_Soc	int (0 no, 1 yes)	Water resources (t)	Water	float (preserves 1 decimal place)
Is there a holiday?	Holiday	int (0 not on holiday, 1 on holiday)	**Personnel factors**		
Activities in region	Act_Reg	int (0 no, 1 yes)	personnel gender	Gender	int (0 female, 1 male)
Working day	Work	int (0 non working day, 1 working day)	Age of personnel	Age	int retains age integers

**Table 2:** Experimental design.

Experimental Subjects	Test Method	Data Type	Comparison of Results
Service hall of Olympic Badminton Stadium	Take a piece of data for a period of time as the data set, randomly select about 70% of the overall data as the training set, and randomly select 30% as the verification set. The digital twin model is used for simulation, and the training set data are used to predict the future lighting, temperature, air supply, and other parameters, and predict the energy consumption change in each period.	1. Main data: Power consumption, temperature and humidity, number of personnel, weather, and equipment startup status. 2. Auxiliary data: Sound, wind speed, data of opening and closing windows, data of opening and closing doors, and activity.	1. The prediction value is compared with the prediction of the validation set and if the accuracy of the overall prediction value is more than 80% then the prediction model is feasible. 2. Set the energy-saving regulation prediction and compare the prediction results with the verification set data again. Find the difference, compare the data of each dimension at this time, and achieve energy-saving improvement measures.

**Table 3:** Sensor selection.

Sensor Name	Type	Function
Temperature and humidity sensor	DHT11	Collect temperature and humidity data in the space.
Vibration sensor	UltiRobot	Detect the vibration in the activity site.
Air Ultrasonic Ceramic Transducers	URM04 RS485	Monitor the distance between the window and the window frame to judge the window opening value.
Light sensor	——	Read indoor and outdoor light intensity.
Sound sensor	——	Monitor sound loudness.
Air flow sensor	——	Monitor air flow.
Human infrared sensor	——	Monitor the flow of people in and out of the door.
Camera	Sentry2 k210	Capture the number of indoor personnel.
Wi-Fi module	ESP8266	Connect all data collection terminals to form an IoT system.
Multi-function electric meters	Three-phase guide rail	Monitor the power consumption of each circuit.

**Table 4:** Event and corresponding probability.

Number	Corresponding Content	Probability of Someone Playing	Probability of Using Site 1
1	Working period	0.33	0.5
2	The outdoor weather is good	0.7	0.3
3	Major badminton competitions are being held	0.1	0.5
4	There are competitions in the school	0.5	0.7
5	Students use probability	0.3	0.3
6	The site environment and relevant equipment are in good condition	0.95	0.7

**Table 5:** Importance of each feature calculated by random forest.

Number	Feature	Influence Weight
0	Maximum temperature of the day	0.359321
1	Minimum temperature of the day	0.229884
2	Weather condition	0.051107
3	Condition of equipment	0.089802
4	Wind level	0.019261
5	Medical protection control	0.059630
6	Whether it is a working day	0.048962
7	Winter vacation or not	0.115273
8	There are related large-scale activities in society	0.010520
9	Campus activities	0.016242

**Table 6:** Results of influencing factors.

Number	Feature	Influence Weight
0	Maximum temperature of the day	0.61
1	Minimum temperature of the day	0.166
2	Weather conditions	0.022
3	Equipment situation	0.086
4	Wind level	0.018
5	Epidemic control	0.045
6	Workday	0.031
7	Is there a holiday	0.007
8	Large social events	0.009
9	On-campus activities	0.007

**Table 7:** Cause mining of abnormal data.

Data Number	Date	Power Consumption (kWh/day)	Predicted Power Consumption (kWh/day)	Error Magnitude	Mean Percentage Error	Abnormal Cause Tracing
10	22 January 2022	267.1	308	40.9	0.153126	Snowy and non-working days
26	7 February 2022	253.2	302	48.8	0.192733	Unknown
33	14 February 2022	457	340	−117	0.256018	Serious equipment anomalies
38	19 February 2022	177	264	87	0.491525	Serious equipment anomalies
47	28 February 2022	232.8	278	45.2	0.194158	Serious equipment anomalies
48	1 March 2022	322.3	272	−50.3	0.156066	Serious equipment anomalies
109	1 May 2022	107.5	129	21.5	0.2	First week of strict epidemic control

## Data Availability

The data used to support the findings of this study are available from the corresponding author upon request.
